# Supplementary material for: Fishing during extreme heatwaves alters ecological interactions and increases indirect fishing mortality in a ubiquitous nearshore system
Source: Commun Biol. 2025 May 12;8:735. doi: 10.1038/s42003-025-08158-w (PMC12069597; doi:10.1038/s42003-025-08158-w)
Supplement: Supplementary file 1 — Supplementary Information [file 42003_2025_8158_MOESM1_ESM.pdf]

Supplementary Information

Supplementary material to: Fishing during extreme heatwaves alters ecological interactions and increases indirect fishing mortality in a ubiquitous nearshore system

Jeff C. Clements[1,2], Sarah Harrison[1,2], Mylène Roussel[1], Jillian Hunt[1], Brooke-Lyn Power[1,2], Rémi Sonier[1]

[1] *Fisheries and Oceans Canada, 343 Université Ave., Moncton, NB E1C 9B6, Canada*

[2] *University of New Brunswick, Department of Biological Sciences, 100 Tucker Park Rd., Saint John, NB E2L 4L5, Canada*

*** Correspondence:** Jeff C. Clements, PhD
Fisheries and Oceans Canada, Gulf Region
343 Université Avenue, Moncton, NB E1C 9B6, Canada
Email: jeffery.clements@dfo-mpo.gc.ca
Tel: +1 (506) 866-6655
ORCID: https://orcid.org/0000-0001-5140-5751

Table of Contents

Supplementary Figures 1
Figure S1........ 2
Figure S2........ 3
Figure S3........ 4
Figure S4........ 5
Figure S5........ 6
Supplementary Tables 7
Table S1. 8
Table S2. 9
Table S3. 10
Table S4. 11
Table S5. 12
Table S6. 13
Table S7. 14
Data Dictionary 15
Preface 16
Supplementary Data 1 17
Supplementary Data 2 18
Supplementary Data 3 19
Supplementary Data 4 20
Supplementary Data 5 21
Supplementary Data 6 22
Supplementary Data 7 23
Supplementary Data 8 24
Supplementary Data 9 25
Supplementary Data 10 26

Supplementary Figures

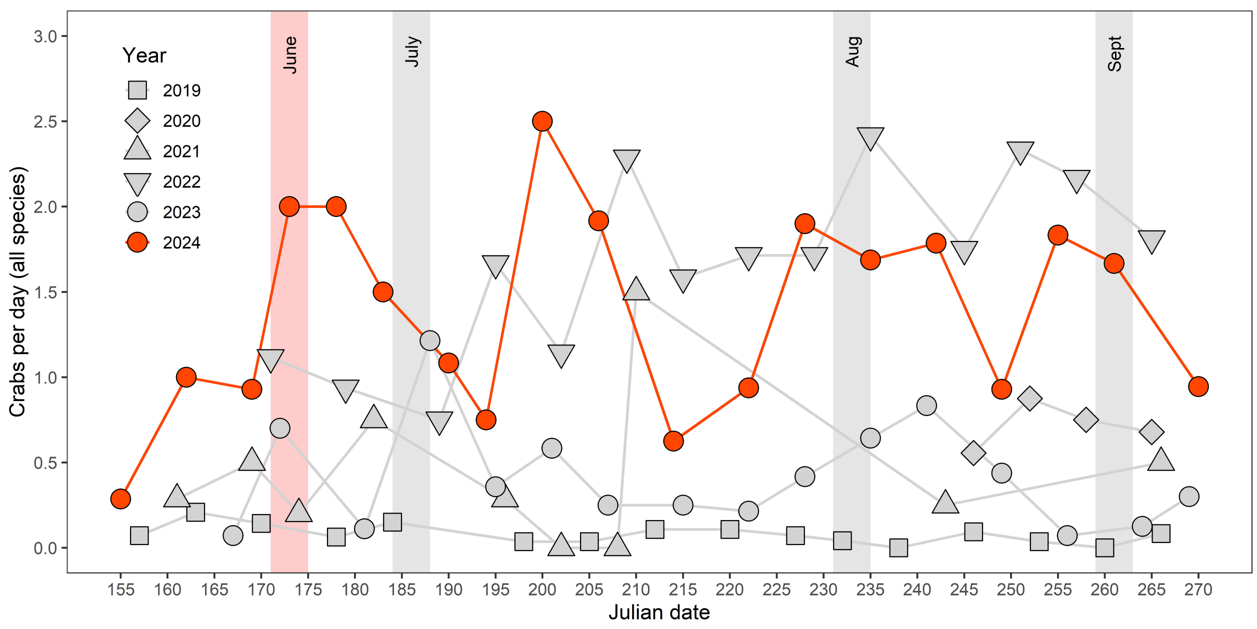

Figure S1. Average crab catches per unit effort at the 'Loggiecroft' monitoring site from the Kouchibouguac crab monitoring program from 2019-2024. Shaded areas in the top panels denote the timing and duration of each of the three-day mesocosm experiments conducted in 2024. Each point represents an average of 1-4 traps within a year (n = 4, 4, 1, 2, 2, 2 sequentially from 2019-2024). Note that the dates of the crab monitoring program did not capture the days of our May experiment.

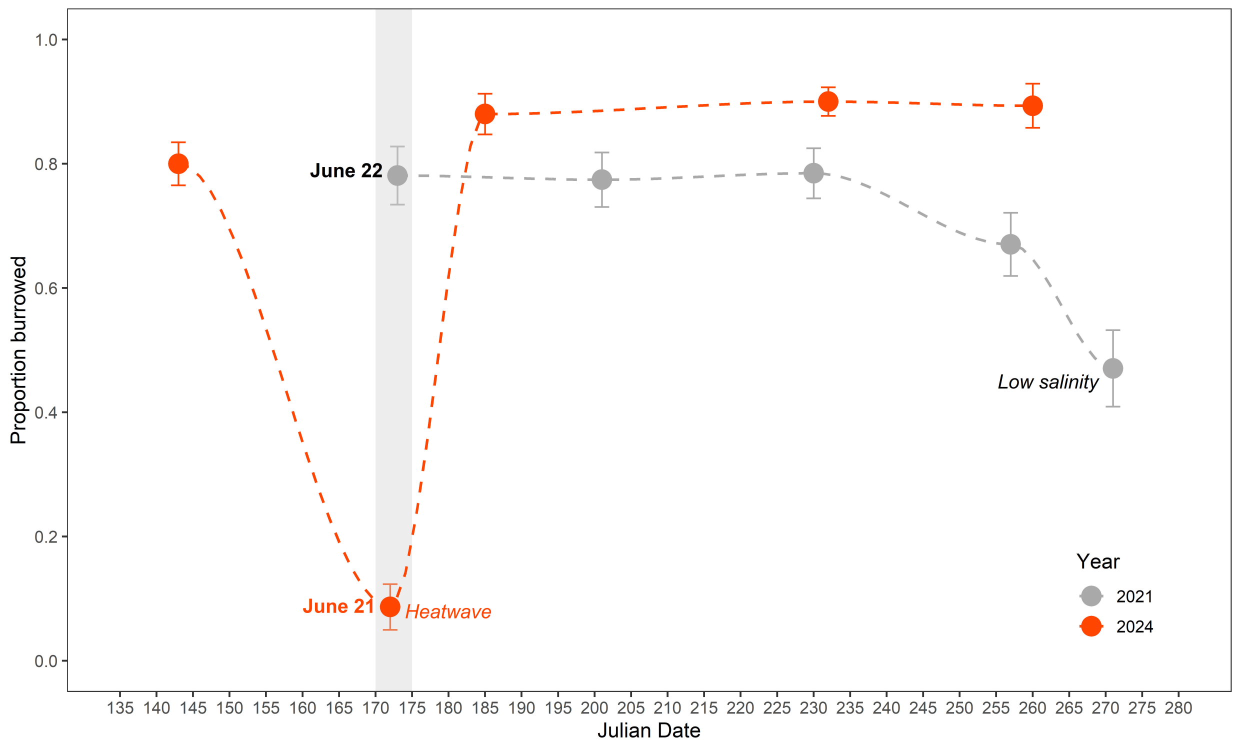

Figure S2. Proportion of sub-legal soft-shell clams reburrowed as a function of Julian date for two separate experiments conducted in 2021 (grey) and 2024 (red) at Kouchibouguac National Park. Data for 2021 are previously unpublished data from experiments detailed in Ledoux et al. (2023; https://doi.org/10.1016/j.jembe.2023.151916). Data for 2024 were collected during the present study. The experimental trails conducted in June for each of the two years are highlighted by the grey shaded area. Acute environmental stress events captured by the two experiments are also denoted (i.e., *Heatwave* and *Low salinity*); the low salinity event in 2021 was driven by heavy rainfall. Dashed lines represent Loess fit curves for each year (0.5 step). Data points for 2021 are mean ± standard error of burrowing proportions for each experimental trial computed from n = 3 clams in each of n= 12 buckets across n = 4 sites in Kouchibouguac National Park (note that some buckets were excluded in 2021 as burrowing could not be determined; see Supplementary Data 7 for raw data and Ledoux et al. (2023) for further explanation on data omissions). Data points for 2024 are mean ± standard error of burrowing proportions for each experimental trial computed from n = 5 clams in each of n = 15 buckets at n = 1 site in Kouchibouguac National Park (no data omissions).

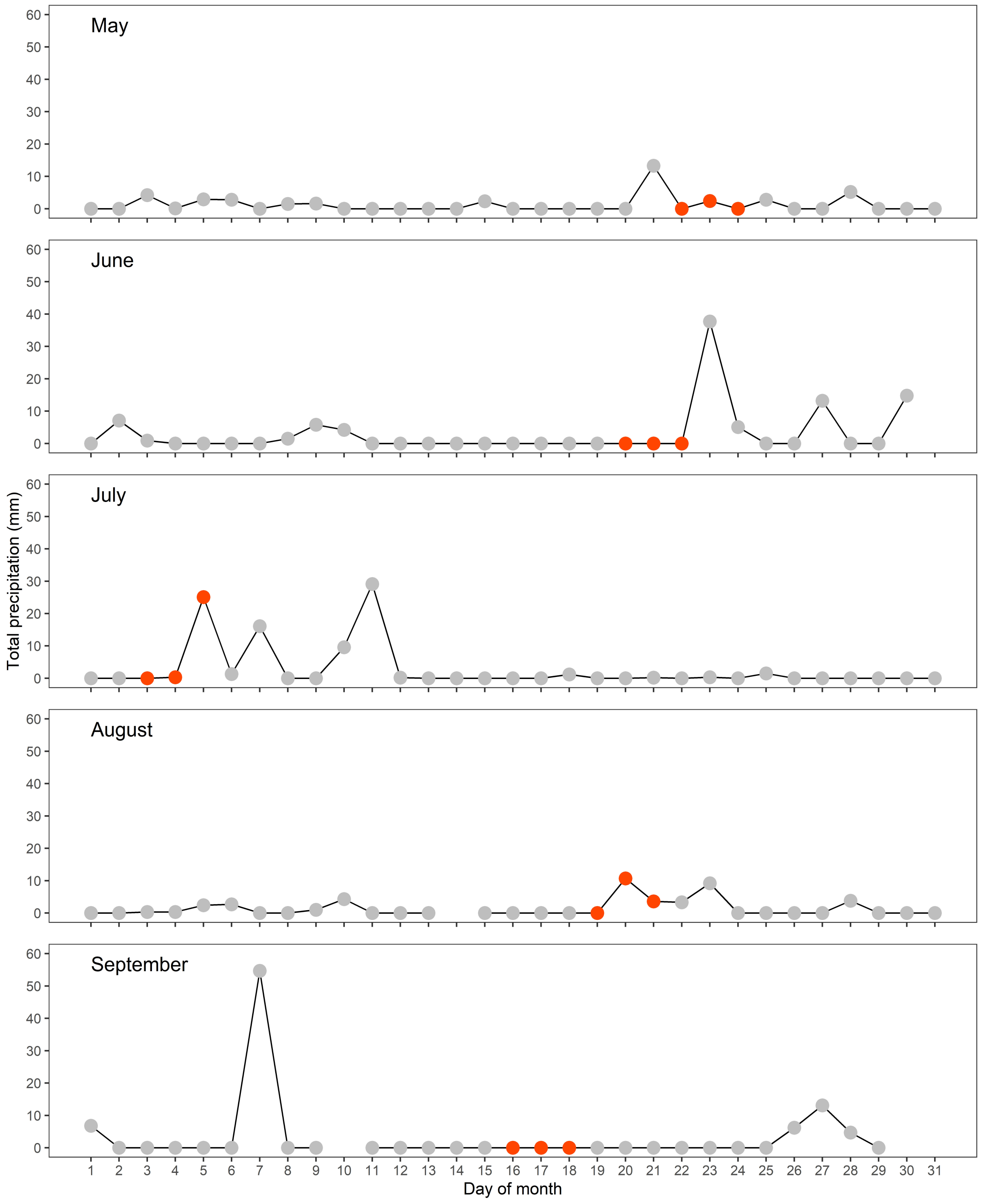

Figure S3. Daily precipitation amounts (in mm) for each of the five months (May-September) in which experimental trials were conducted. Red points denote the three days during which the experimental trials took place in each month. Precipitation data were obtained from Environment Canada (Kouchibouguac station; https://climate.weather.gc.ca/).

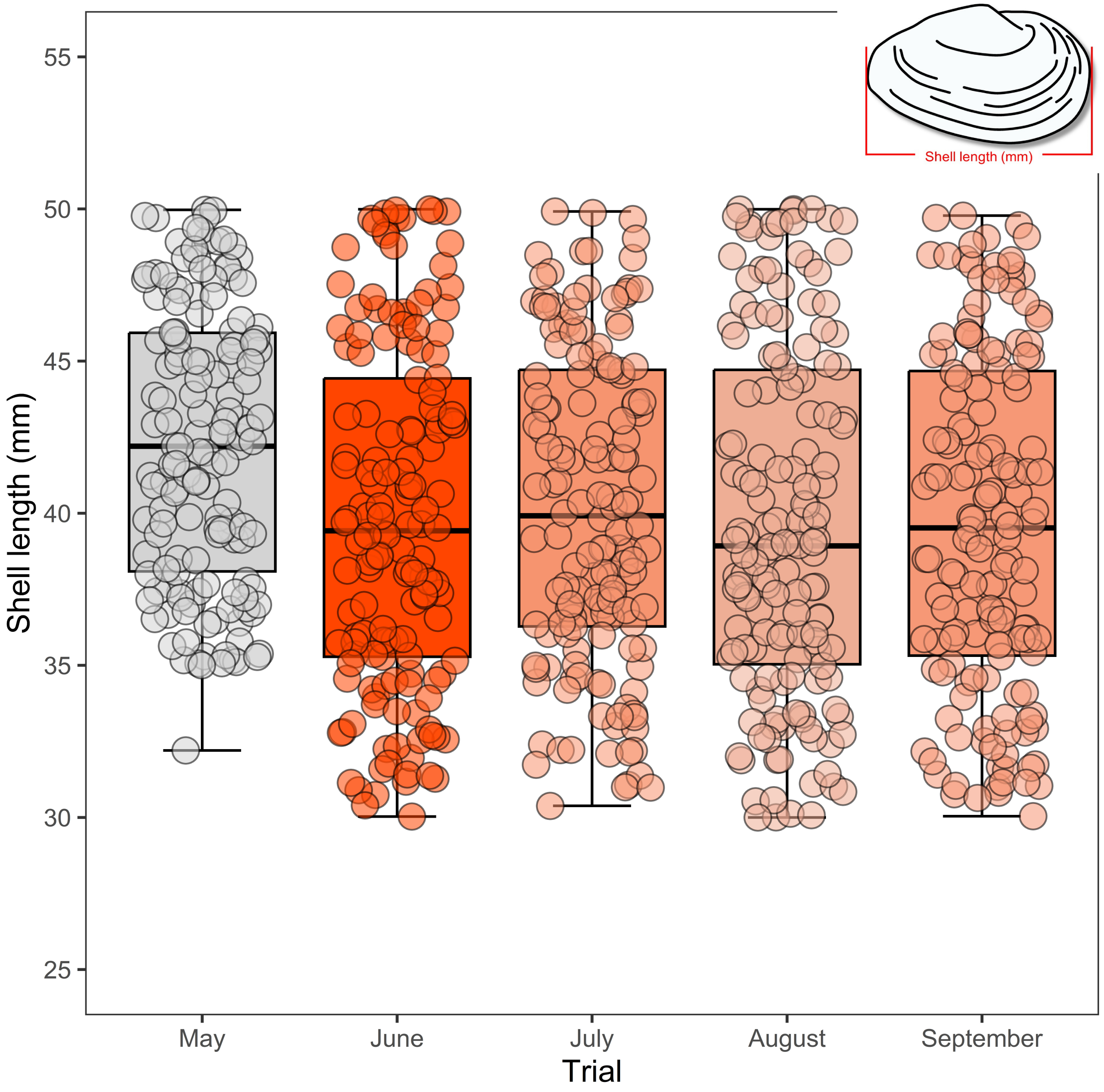

Figure S4. Size (shell length, mm) distributions of experimental sub-legal clams during each experimental trial (n = 150 clams per experiment). Colours denote a gradient from lowest (gray) to highest (red) mean air temperature during fishing.

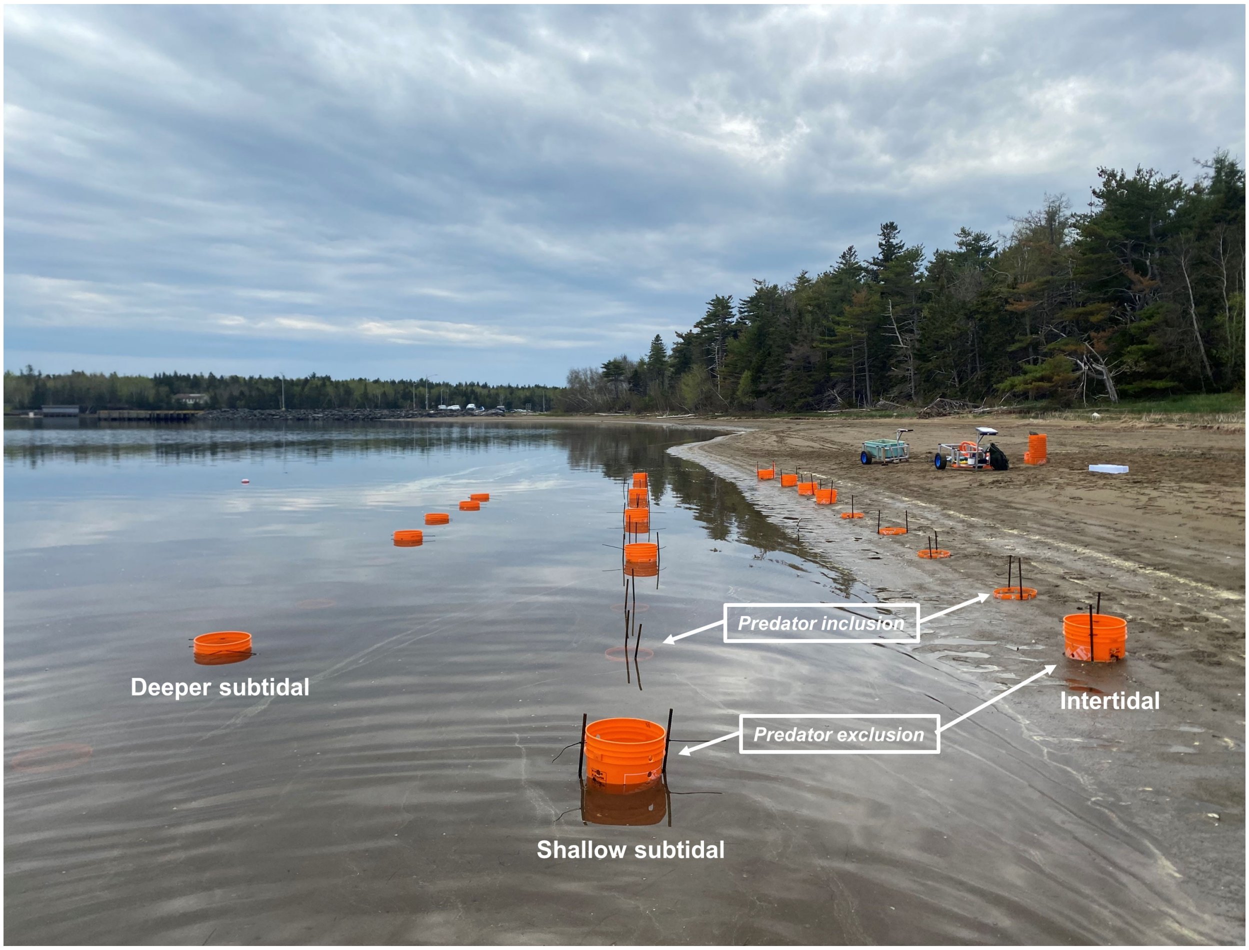

Figure S5. Image of the experimental layout during the May experiment showing the three tide levels and different predator treatments. Not all replicate plots are visible in the photo (actual replication: n = 5 plots per predator treatment × tide level × experiment combination). Also note that lids are not on the predator exclusion plots in this photo (photo taken before clams were placed in plots). The spatial placement of predator treatments was randomized prior to each experimental trial.

Supplementary Tables

Table S1. Results of Bayesian generalized linear mixed models for the effects of experimental trial (May, June, July, August, September), predator treatment (predator inclusion, predator exclusion), time since fishing (24 h, 48 h), and tide level (intertidal, shallow subtidal, deeper subtidal) on the proportion of reburowed and dead clams. Statistical models included Plot ID as a random variable to account for spatial effects and repeated measures over the two time points. Results were generated using the *Anova()* function from the 'car' package in R, which provides Wald chi-square test results for fixed effects. Bolded text denotes significant effects at p ≤ 0.05; italicized text denotes marginally non-significant effects at p ≤ 0.10.

Source of error	χ^2	df	p-value
Reburrowing			
**(Intercept)**	**7.75**	**1**	**0.0054**
**Trial**	**16.13**	**4**	**0.0029**
Predator treatment	0.01	1	0.9055
Time	0.04	1	0.8502
Tide level	1.17	2	0.5585
Trial × Predator treatment	3.61	4	0.4618
Trial × Time	1.60	4	0.8094
Predator treatment × Time	0.03	1	0.8529
Trial × Tide level	1.78	8	0.9870
Predator treatment × Tide level	0.02	2	0.9909
Time × Tide level	1.35	2	0.5104
Trial × Predator treatment × Time	0.59	4	0.9642
Trial × Predator treatment × Tide level	0.89	8	0.9989
Trial × Time × Tide level	0.64	8	0.9997
Predator treatment × Time × Tide level	0.37	2	0.8295
Trial × Predator treatment × Time × Tide level	0.33	8	>0.9999
Mortality			
**(Intercept)**	**44.14**	**1**	**<0.0001**
**Trial**	**37.07**	**4**	**<0.0001**
**Predator treatment**	**11.53**	**1**	**0.0007**
Time	*3.40*	*1*	*0.0651*
Tide level	0.64	2	0.7264
Trial × Predator treatment	3.06	4	0.5474
**Trial × Time**	**12.80**	**4**	**0.0123**
**Predator treatment × Time**	**9.03**	**1**	**0.0027**
Trial × Tide level	5.46	8	0.7072
Predator treatment × Tide level	1.20	2	0.5487
Time × Tide level	4.18	2	0.1239
Trial × Predator treatment × Time	*8.40*	*4*	*0.0781*
Trial × Predator treatment × Tide level	8.74	8	0.3646
Trial × Time × Tide level	5.35	8	0.7193
Predator treatment × Time × Tide level	2.58	2	0.2752
Trial × Predator treatment × Time × Tide level	4.05	8	0.8527

Table S2. Results of pairwise tests between experimental trials on the proportion of reburrowed clams. Results were generated using the *pairs()* function from a pairwise model generated using the *emmeans()* function from the 'emmeans' package in R. Bolded text denotes significant effects at $p \leq 0.05$; italicized text denotes marginally non-significant effects at $p \leq 0.10$. Results are pooled across predator treatments, times, and tide levels.

Contrast	Estimate	SE	df	z-ratio	p-value
Aug - July	-1.43	1.32	Inf	-1.08	0.8154
**Aug - June**	**8.19**	**1.93**	**Inf**	**4.24**	**0.0002**
Aug - May	-1.43	1.42	Inf	-1.00	0.8544
Aug - Sept	-0.81	1.15	Inf	-0.70	0.9560
**July - June**	**9.62**	**2.22**	**Inf**	**4.34**	**0.0001**
July - May	0.01	1.80	Inf	0.01	>0.9999
July - Sept	0.63	1.61	Inf	0.39	0.9952
**June - May**	**-9.62**	**2.24**	**Inf**	**-4.28**	**0.0002**
**June - Sept**	**-9.00**	**2.13**	**Inf**	**-4.23**	**0.0002**
May - Sept	0.62	1.68	Inf	0.37	0.9961

Table S3. Results of pairwise tests between experimental trials for each predator treatment and each time (since fishing) for the proportion of dead clams. Results were generated using the *pairs()* function from a pairwise model generated using the *emmeans()* function from the 'emmeans' package in R. Bolded text denotes significant effects at p ≤ 0.05; italicized text denotes marginally non-significant effects at p ≤ 0.10. Results are pooled across tide levels.

Contrast	Estimate	SE	df	z-ratio	p-value
Predator exclusion					
24 h:					
Aug - July	-0.32	2.99	Inf	-0.11	>0.9999
**Aug - June**	**-10.99**	**2.05**	**Inf**	**-5.36**	**<0.0001**
Aug - May	4.56	3.29	Inf	1.39	0.6368
Aug - Sept	-1.74	2.71	Inf	-0.64	0.9683
**July - June**	**-10.67**	**3.58**	**Inf**	**-2.98**	**0.0239**
July - May	4.87	4.44	Inf	1.10	0.8075
July - Sept	-1.42	4.01	Inf	-0.36	0.9966
**June - May**	**15.54**	**3.87**	**Inf**	**4.02**	**0.0006**
**June - Sept**	**9.25**	**3.11**	**Inf**	**2.97**	**0.0247**
May - Sept	-6.29	4.25	Inf	-1.48	0.5756
48 h:					
Aug - July	-0.47	3.53	Inf	-0.13	0.9999
**Aug - June**	**-18.41**	**2.85**	**Inf**	**-6.46**	**<0.0001**
Aug - May	4.93	4.34	Inf	1.14	0.7877
Aug - Sept	-2.01	3.59	Inf	-0.56	0.9809
**July - June**	**-17.93**	**4.46**	**Inf**	**-4.02**	**0.0006**
July - May	5.40	5.57	Inf	0.97	0.8690
July - Sept	-1.54	5.00	Inf	-0.31	0.9981
**June - May**	**23.33**	**5.17**	**Inf**	**4.51**	**0.0001**
**June - Sept**	**16.40**	**4.54**	**Inf**	**3.61**	**0.0028**
May - Sept	-6.93	5.61	Inf	-1.24	0.7298
Predator inclusion					
24 h:					
Aug - July	2.32	3.43	Inf	0.68	0.9617
**Aug - June**	**-9.01**	**2.22**	**Inf**	**-4.06**	**0.0005**
Aug - May	9.03	4.33	Inf	2.08	0.2275
Aug - Sept	-3.56	2.26	Inf	-1.58	0.5117
**July - June**	**-11.33**	**3.97**	**Inf**	**-2.86**	**0.0349**
July - May	6.71	5.50	Inf	1.22	0.7405
July - Sept	-5.88	3.99	Inf	-1.47	0.5792
**June - May**	**18.03**	**4.85**	**Inf**	**3.72**	**0.0019**
**June - Sept**	**5.45**	**1.68**	**Inf**	**3.24**	**0.0104**
May - Sept	*-12.58*	*4.86*	*Inf*	*-2.59*	*0.0722*
48 h:					
Aug - July	0.78	1.62	Inf	0.48	0.9888
Aug - June	-9.82	4.05	Inf	-2.42	0.1089
Aug - May	9.11	5.14	Inf	1.77	0.3892
Aug - Sept	1.09	1.75	Inf	0.62	0.9715
July - June	*-10.61*	*4.25*	*Inf*	*-2.50*	*0.0918*
July - May	8.33	5.27	Inf	1.58	0.5111
July - Sept	0.31	2.04	Inf	0.15	0.9999
**June - May**	**18.93**	**6.50**	**Inf**	**2.91**	**0.0294**
June - Sept	*10.91*	*4.27*	*Inf*	*2.56*	*0.0790*
May - Sept	-8.02	5.32	Inf	-1.51	0.5567

Table S4. Results of pairwise tests between predator treatments for each experimental trial and time (since fishing) for the proportion of dead clams. Results were generated using the *pairs()* function from a pairwise model generated using the *emmeans()* function from the 'emmeans' package in R. Bolded text denotes significant effects at $p \leq 0.05$; italicized text denotes marginally non-significant effects at $p \leq 0.10$. Results are pooled across tide levels.

Contrast	Estimate	SE	df	z-ratio	p-value
24 h					
May: PE - PI	-1.23	3.88	Inf	-0.32	0.7512
**June: PE - PI**	**-3.72**	**1.13**	**Inf**	**-3.29**	**0.0010**
July: PE - PI	-3.06	3.60	Inf	-0.85	0.3943
**Aug: PE - PI**	**-5.70**	**2.07**	**Inf**	**-2.75**	**0.0059**
**Sept: PE - PI**	**-7.52**	**3.11**	**Inf**	**-2.42**	**0.0157**
48 h					
May: PE - PI	*-8.46*	*5.1*	*Inf*	*-1.66*	*0.0971*
June: PE - PI	-4.06	4.01	Inf	-1.01	0.3109
**July: PE - PI**	**-11.39**	**4.38**	**Inf**	**-2.60**	**0.0093**
**Aug: PE - PI**	**-12.65**	**2.74**	**Inf**	**-4.62**	**<0.0001**
**Sept: PE - PI**	**-9.55**	**4.46**	**Inf**	**-2.14**	**0.0325**

Table S5. Results of pairwise tests between individual time points (since fishing) for each experimental trial and predator treatment for the proportion of dead clams. Results were generated using the *pairs()* function from a pairwise model generated using the *emmeans()* function from the 'emmeans' package in R. Bolded text denotes significant effects at $p \leq 0.05$; italicized text denotes marginally non-significant effects at $p \leq 0.10$. Results are pooled across tide levels.

Contrast	Estimate	SE	df	z-ratio	p-value
Predator exclusion					
May: 24 - 48 h	3.11	3.96	Inf	0.78	0.4329
**June: 24 - 48 h**	**-4.68**	**1.69**	**Inf**	**-2.78**	**0.0055**
July: 24 - 48 h	2.58	3.69	Inf	0.70	0.4837
Aug: 24 - 48 h	*-4.48*	*2.38*	*Inf*	*-1.88*	*0.0605*
Sept: 24 - 48 h	2.47	3.35	Inf	0.74	0.4616
Predator inclusion					
May: 24 - 48 h	-4.13	4.8	Inf	-0.86	0.3898
June: 24 - 48 h	-5.03	3.98	Inf	-1.26	0.2067
July: 24 - 48 h	-5.75	3.92	Inf	-1.47	0.1427
**Aug: 24 - 48 h**	**-4.21**	**2.12**	**Inf**	**-1.99**	**0.0472**
Sept: 24 - 48 h	0.44	1.91	Inf	0.23	0.8183

Table S6. Results of generalized additive mixed models for the effects of average air temperature during fishing (continuous smoothed term), predator treatment (categorical parametric term with two levels; predator inclusion, predator exclusion), time since fishing (categorical parametric term with two levels; 24 h, 48 h), and tide level (categorical parametric term with three levels; intertidal, shallow subtidal, deeper subtidal) on the proportion of reburrowed and dead clams. Interactions between the three categorical parametric terms were included in the models. Statistical models included Julian date (continuous smoothed term) as a random variable. Results were generated using the *anova()* function, specifying '$gam' on the model (built using the *gam()* function from the 'mgcv' package). Bolded text denotes significant effects at p ≤ 0.05; italicized text denotes marginally non-significant effects at p ≤ 0.10. For smooth terms, 'edf' = effective degrees of freedom;'rdf' = reference degrees of freedom.

Term type	Source of error	df (edf, rdf)	F-value	P-value
Reburrowing				
Parametric	Predator treatment	1	1.7	0.1870
	Time	1	0.1	0.7830
	Tide level	2	1.3	0.2860
	Predator treatment × Time	1	0.1	0.8050
	Predator treatment × Tide level	2	0.1	0.9220
	Time × Tide level	2	0.2	0.8160
	Predator treatment × Time × Tide level	2	0.0	0.9760
Smooth	**Average air temperature**	**(2.0, 2.0)**	**164.4**	**<0.0001**
	**Julian date**	**(5.4, 1.0)**	**0.0**	**0.0137**
Mortality				
Parametric	**Predator treatment**	**1**	**12.5**	**0.0005**
	**Time**	**1**	**8.8**	**0.0032**
	Tide level	2	1.4	0.2610
	**Predator treatment × Time**	**1**	**5.1**	**0.0252**
	Predator treatment × Tide level	2	1.8	0.1668
	Time × Tide level	2	1.1	0.3305
	Predator treatment × Time × Tide level	2	1.2	0.3123
Smooth	**Average air temperature**	**(1.0, 1.0)**	**202.5**	**<0.0001**
	Julian date	(3.0, 1.0)	0.0	0.2790

Table S7. Approximate timing (hh:mm) of events for each of the five experimental trials, including the predicted low tide time at Pointe Sapin (Environment Canada Tide Station 01830), the approximate time that fishing commenced (time of first shell length recording), trial start time, and the maximum time that a clam would have endured handling from the time it was captured to the time it was released into the experimental plot. Note that the "Fishing to release" time represents the time from the first clam being caught and measured to the time the experiment started.

Trial	Date	Low tide	Fishing start	Trial start	Fishing to release
May	22-May	11:36	10:50	14:15	3:25
June	20-Jun	11:38	10:20	14:25	4:05
July	3-Jul	10:13	8:45	13:15	4:30
August	19-Aug	11:41	11:35	15:45	4:10
September	16-Sep	9:54	9:30	14:00	4:30

Data Dictionary

Preface

This data dictionary is provided to facilitate understanding and transparency for our open datasets (Supplementary Data 1-10) that were used to statistically analyze data and generate figures in the main paper, supplementary figures and tables, and supplementary analysis. The datasets are provided in separate tabs in a single .xlsx file in the Electronic Supplementary Material alongside the online version of this article. Raw csv files can also be accessed through the Government of Canada's Open Government Portal at https://open.canada.ca/data/dataset/1bf057da-8280-11ef-8cce-55cc7f028297. In the following pages, we generally describe the nature of each dataset, specify which analyses and/or figures each dataset pertains to, and provide written descriptions for each column header. Further questions about the data can be directed to Dr. Jeff Clements at jeffery.clements@dfo-mpo.gc.ca

Supplementary Data 1

Description: Reburrowing and mortality data for individual mesocosm plots broken down by levels of the experimental independent variables for 24- and 48 h after fishing.

Pertains to: Statistical analysis in main paper (BGLMM + GAMM analyses)
Figure 2

Column name	Description
month	Categorical fixed independent variable with five levels (May, June, July, Aug, Sept). The month that each experimental trial was conducted in. Simply referred to as "Experiment" throughout the main paper.
time.since.deploy	Categorical fixed independent variable with two levels (24 h, 48 h). The time point at which the number of reburrowed and dead clams was recorded after fishing in their respective mesocosm plots.
tide.level	Categorical fixed independent variable with three levels (intertidal, shallow subtidal, deeper subtidal). The tide level at which the clams were released into their respective mesocosm plots. IN = intertidal; S1 = shallow subtidal; S2 = deeper subtidal.
plot.glob	Categorical random independent variable with 150 levels. The identification of each individual mesocosm plot within each experimental trial. Individual plots are repeated twice within each experiment, as the exact same plot was assessed twice in each experimental trial. Referred to as "Plot ID" in the main paper.
pred.treat	Categorical fixed independent variable with two levels (predator exclusion, predator inclusion). The type of mesocosm plot used with respect to including or excluding crab predators. PE = predator exclusion; PI = predator inclusion.
initial	The number of clams initially placed on the sediment within each mesocosm plot (this number is always 5).
burrowed	The number of clams that reburrowed in each individual mesocosm plot at each time point.
dead	The number of clams that died in each individual mesocosm plot at each time point.
prop.burrowed	The proportion of clams that reburrowed in each individual mesocosm plot at each time point. Computed as: *prop.burrowed = burrowed ÷ initial*
prop.dead	The proportion of clams that died in each individual mesocosm plot at each time point. Computed as: *prop.dead = dead ÷ initial*

Supplementary Data 2

Description: Daily temperature (in ºC) time series data obtained from historical weather data published by Environment and Climate Change Canada for the weather station closest to our study site, Kouchibouguac (URL: https://climate.weather.gc.ca/historical_data/search_historic_data_stations_e.html?StationID=26968&Year=2018&Month=3&Day=5&timeframe=1&StartYear=1840&EndYear=2020&type=line&MeasTypeID=dptemp&time=LST&searchType=stnProx&txtRadius=25&optProxType=navLink&txtLatDecDeg=46.783333333333&txtLongDecDeg=65.016666666667&optLimit=specDate&selRowPerPage=25&station=KOUCHIBOUGUAC+CS).

Pertains to: Figure 1 (time series data in top two panels)

Column name	Description
date	The date of the temperature record in MM/DD/YYYY format.
julian date	The Julian date corresponding to the date of the temperature record.
max.temp	The maximum hourly temperature recorded for a given date.
mean.temp	The average temperature for a given date, taken from hourly temperature readings (n = 24).

Supplementary Data 3

Description: Hourly air temperature and humidex values (in ºC) during the approximate four hours in which clams were fished in each of the five experimental trials. Data were obtained from historical weather data published by Environment and Climate Change Canada for the weather station closest to our study site, Kouchibouguac (URL: https://climate.weather.gc.ca/historical_data/search_historic_data_stations_e.html?StationID=26968&Year=2018&Month=3&Day=5&timeframe=1&StartYear=1840&EndYear=2020&type=line&MeasTypeID=dptemp&time=LST&searchType=stnProx&txtRadius=25&optProxType=navLink&txtLatDecDeg=46.783333333333&txtLongDecDeg=65.016666666667&optLimit=specDate&selRowPerPage=25&station=KOUCHIBOUGUAC+CS).

Pertains to: Figure 1 (trial-specific data in bottom two panels)

Column name	Description
date	The date of the temperature records in MM/DD/YYYY format.
month	The month that each experiment was conducted in. Simply referred to as "experimental trial" or "trial" throughout the main paper.
hour	The numerical hour of the temperature record
temp	The air temperature value for the corresponding hour on the corresponding date
humidex	The humidex value for the corresponding hour on the corresponding date

Supplementary Data 4

Description: Estimates of predator activity during each experimental trial.

Pertains to: Figure 3
Figure 4 ('Relative predator activity' curve)

Column name	Description
month	The month that each experimental trial was conducted in. Simply referred to as "Experiment" throughout the main paper.
avg.air.temp	Average air temperature during the approximate four hours in which clams were fished in each of the five experimental trials (i.e., average of hourly air temperatures in S3 Data)
crab.count	The number of live crabs within each individual mesocosm plot at each time point. Crab species observed in mesocosms included invasive European green crabs (*Carcinus maenas*) and native rock crabs (*Cancer irroratus*).
mudsnail.buckets.count	The number of individual mesocosm plots that contained multiple native mudsnails, *Ilyanassa obsoleta*.
tot.pred.activity	Index of total predator activity for each experiment, computed as: *tot.pred.activity = crab.count + mudsnail.bucket.count*
rel.pred.activity	Index of predator activity for each experiment relative (i.e., proportional) to the highest *tot.pred.activity* value observed (June trial = 42), Computed as: *rel.pred.activity = tot.pred.activity ÷ 42*

Supplementary Data 5

Description: Reburrowing and mortality proportions for individual mesocosm plots at 48 h after fishing in relation to the mean air temperature during the approximate four hours in which clams were fished in each of the five experimental trials (i.e., average of hourly air temperatures for each trial in S-03 Data).

Pertains to: Figure 4 (‘Reburrowing’ and ‘Mortality’ curves)

**Column name**	**Description**
month	The month that each experimental trial was conducted in. Simply referred to as “experimental trial” or “trial” throughout the main paper.
exp.temp	Average air temperature during the approximate four hours in which clams were fished in each of the five experimental trials (i.e., average of hourly air temperatures in S3 Data)
metric	The endpoint recorded in reference to the clams. burrowed.48h = clams reburrowed after 48 h; dead.48h = clams dead after 48 h.
proportion	The numerical proportion of clams either reburrowed (*metric*: burrowed.48h) or dead (*metric*: dead.48h) for each mesocosm plot after 48 h.

Supplementary Data 6

Description: Crab catch data from the crab monitoring program at Kouchibouguac National Park from 2019-2024.

Pertains to: Figure S1

Column name	Description
year	The year of data collection for the monitoring program.
julian.date	The Julian date corresponding to the date of data collection within each year.
days.fished	Number of days traps were deployed for fishing crabs.
avg.mucrab	Average mud crab (*Panopeus herbstii*) catch for a given sampling day within a given year. Computed as: *mud crab catch ÷ number of traps deployed*
avg.rockcrab	Average rock crab (*Cancer irroratus*) catch for a given sampling day within a given year. Computed as: *rock crab catch ÷ number of traps deployed*
avg.rgreencrab	Average green crab (*Carcinus maenas*) catch for a given sampling day within a given year. Computed as: *green crab catch ÷ number of traps deployed*
avg.total.crab	Average catch of all crab species for a given sampling day within a given year. Computed as: *total crab catch ÷ number of traps deployed*
avg.cpue	The average catch per unit effort for all species of crabs for a given sampling day within a given year. Computed as: *avg.total.crab ÷ number of days traps were deployed*

Supplementary Data 7

Description: Reburrowing proportion data (24 h after sub-legal soft-shell clams were tossed back) for comparing temporal shifts in reborrowing between the mesocosm experiment presented in the paper and a comparable experiment conducted in 2021 (Ledoux et al. 2021; https://doi.org/10.1016/j.jembe.2023.151916).

Pertains to: Figure S2

Column name	Description
year	The year of the experiment.
date	The calendar date corresponding to the date of data collection within each year.
day	The Julian date corresponding to the date of data collection within each year.
site	The site within Kouchibouguac National Park that experiments were conducted (n = 4 for 2021; n = 1 for 2024). Specifics on sites for 2021 can be found in Ledoux et al. (2021) here: https://doi.org/10.1016/j.jembe.2023.151916
bucket	Individual experimental (mesocosm) plot ID.
released	Number of sub-legal clams released back to the sediment after fishing.
burrowed	Number of sub-legal clams that were completely reborrowed 24 hours after release.
prop.burrowed	The proportion of clams that reburrowed in each individual mesocosm plot at each time point. Computed as: *prop.burrowed = burrowed ÷ released*

Supplementary Data 8

Description: Daily precipitation values (May 1-September 30, 2024) obtained from historical weather data published by Environment and Climate Change Canada for the weather station closest to our study site, Kouchibouguac (URL: https://climate.weather.gc.ca/historical_data/search_historic_data_stations_e.html?StationID=26968&Year=2018&Month=3&Day=5&timeframe=1&StartYear=1840&EndYear=2020&type=line&MeasTypeID=dptemp&time=LST&searchType=stnProx&txtRadius=25&optProxType=navLink&txtLatDecDeg=46.783333333333&txtLongDecDeg=65.016666666667&optLimit=specDate&selRowPerPage=25&station=KOUCHIBOUGUAC+CS).

Pertains to: Figure S3

Column name	Description
date	The date for which the precipitation data pertains.
month	The month of each date.
day.in.month	The consecutive day within each month (ranging from 1-31).
julian.date	The Julian date corresponding to each date.
precip.mm	The total precipitation amount (in mm) recorded during each day.
trial	Binary variable denoting whether experimental trials were conducted on a given day (N = no trials conducted; E = trials conducted).

Supplementary Data 9

Description: Shell length (in mm) of each individual clam used in each of the five experimental trials.

Pertains to: Figure S4

Column name	Description
experiment	The month that each experimental trial was conducted in. Simply referred to as “experimental trial” or “trial” throughout the main paper.
clam	Identification number assigned to each individual clam, numbered sequentially in the order in which clams were measured for shell length.
shell.length	The shell length (in mm) of each individual clam.

Supplementary Data 10

Description: Reburrowing data for individual mesocosm plots broken down by levels of the experimental independent variables for the 15 min observations conducted two hours after clams were placed on the sediment surface in their respective mesocosm plots on the first day of each experimental trial.

Pertains to: Supplementary analysis
Figure SA1

Column name	Description
month	Categorical fixed independent variable with five levels (May, June, July, Aug, Sept). The month that each experimental trial was conducted in. Simply referred to as "experimental trial" or "trial" throughout the Supplementary analysis.
time.since.deploy	Categorical fixed independent variable with eight levels (15, 30, 45, 60, 75, 90, 105, and 120 mins). The time point at which the number of reburrowed clams was recorded after fishing in their respective mesocosm plots.
tide.level	Categorical fixed independent variable with three levels (intertidal, shallow subtidal, deeper subtidal). The tide level at which the clams were released into their respective mesocosm plots. IN = intertidal; S1 = shallow subtidal; S2 = deeper subtidal.
plot.glob	Categorical random independent variable with 150 levels. The identification of each individual mesocosm plot within each experimental trial. Individual plots are repeated eight time within each trial, as the exact same plot was assessed every 15 mins for two hours. Referred to as "Plot ID" in the Supplementary analysis.
pred.treat	Categorical fixed independent variable with two levels (predator exclusion, predator inclusion). The type of mesocosm plot used with respect to including or excluding crab predators. PE = predator exclusion; PI = predator inclusion.
initial	The number of clams initially placed on the sediment within each mesocosm plot (this number is always 5).
burrowed	The number of clams that reburrowed in each individual mesocosm plot at each time point.
prop.burrowed	The proportion of clams that reburrowed in each individual mesocosm plot at each time point. Computed as: *prop.burrowed = burrowed ÷ initial*
