# Supplementary material for: Fishing during extreme heatwaves alters ecological interactions and increases indirect fishing mortality in a ubiquitous nearshore system
Source: Commun Biol. 2025 May 12;8:735. doi: 10.1038/s42003-025-08158-w (PMC12069597; doi:10.1038/s42003-025-08158-w)
Supplement: Supplementary file 2 — Supplementary Note [file 42003_2025_8158_MOESM2_ESM.pdf]

Supplementary Note

Supplementary material to: Fishing during extreme heatwaves alters ecological interactions and increases indirect fishing mortality in a ubiquitous nearshore system

Jeff C. Clements[1,2]*, Sarah Harrison[1,2], Mylène Roussel[1], Jillian Hunt[1], Brooke-Lyn Power[1,2], Rémi Sonier[1]

[1] *Fisheries and Oceans Canada, 343 Université Ave., Moncton, NB E1C 9B6, Canada*

[2] *University of New Brunswick, Department of Biological Sciences, 100 Tucker Park Rd., Saint John, NB E2L 4L5, Canada*

*** Correspondence:** Jeff C. Clements, PhD
Fisheries and Oceans Canada, Gulf Region
343 Université Avenue, Moncton, NB E1C 9B6, Canada
Email: jeffery.clements@dfo-mpo.gc.ca
Tel: +1 (506) 866-6655
ORCID: https://orcid.org/0000-0001-5140-5751

Background

As mentioned in the main paper, we were interested in longer-term (24-48 h) trends in reburrowing and mortality and thus focus the presentation on those data. Nonetheless, our methodology captured reburrowing rates of tossed back sub-legal clams every 15 mins for two hours following fishing during each experiment, which we feel are worthy of analysis and discussion. As such, we include this supplementary analysis to document initial burrowing rates of sub-legal sized clams after being fished and tossed back on the sediment surface.

Statistical methodology

To briefly reiterate our experimental methodology here, we recorded the proportion of reburrowed clams in each mesocosm plot (at each tide level, and predator treatment during each experiment), every 15 minutes for two hours after "tossing back" the fished sub-legal clams (i.e., placing the clams in the mesocosm plots). As such, the statistical model structure was similar to the analysis in the main paper with two general exceptions: 1. The "time since fishing" factor contained more levels (8 levels: 15, 30, 45, 60, 75, 90, 105, and 120 mins); and 2. we ignored predator treatment, as we did not observe any predators in the mesocosms during this time frame and we would not expect the structure of the different predator treatment plots to affect burrowing. Thus, our model structure had three fixed factors: experiment (5 levels: May, June, July, August, and September), tide level (3 levels: intertidal, shallow subtidal, deeper subtidal), and time since fishing (8 levels, as above). As with the analysis in the main paper, the presence of all-zero observations in some treatment levels resulted in near-complete separation. We thus took the same Bayesian generalized linear mixed modelling approach as described in the main paper. Briefly, models were constructed using the *bglmer()* function the ('blme' package[1]), including plot ID as a categorical random variable to account for random spatial effects and repeated measures across the eight time points, and specifying weak zero-mean normal priors (6,120) to account for near-complete separation (as per Bolker[2]). The lower variance prior was chosen due to the removal of predator treatment as a fixed factor and the increased number of replicates for each fixed factor combination level; the diagonal matrix value of 120 was chosen to match the number of terms in the models. After model construction, the *Anova()* function ('car' package[3]) was then used to obtain model results, using a Type 3 test.

Results and interpretation

The global BGLMM model revealed a significant experiment × time since fishing × tide level effect on the proportion of clams that reburrowed on Day 1 (χ^2_{56} = 109.24, p < 0.0001) (**Fig. SA1, Table SA1**). Given the multiplicative complexity of this interaction, we elected to forego statistical pairwise comparisons and simply discuss general comparisons between experiments, time, and tidal levels, with reference to overlapping error bars for interpretation.

With the exception of four clams in the September experiment, clams in the intertidal plots were unable to reburrow within two hours of being tossed back (**Fig. SA1**). This coincides with the results of previous studies[4] and reiterates that clams require submergence in water in order to burrow[5]. As for the four exceptional intertidal clams that were fully burrowed at the intertidal level in September: we cannot functionally explain these occurrences from our observations. It is

perhaps possible that these individuals were captured and deployed in the intertidal in prior experiments and perhaps had habituated to such conditions, as bivalves are reported to be able to habituate to repeated stimuli despite being uncephalized[6,7]. Regardless, this finding speaks to the incredible functional diversity exhibited by marine bivalves.

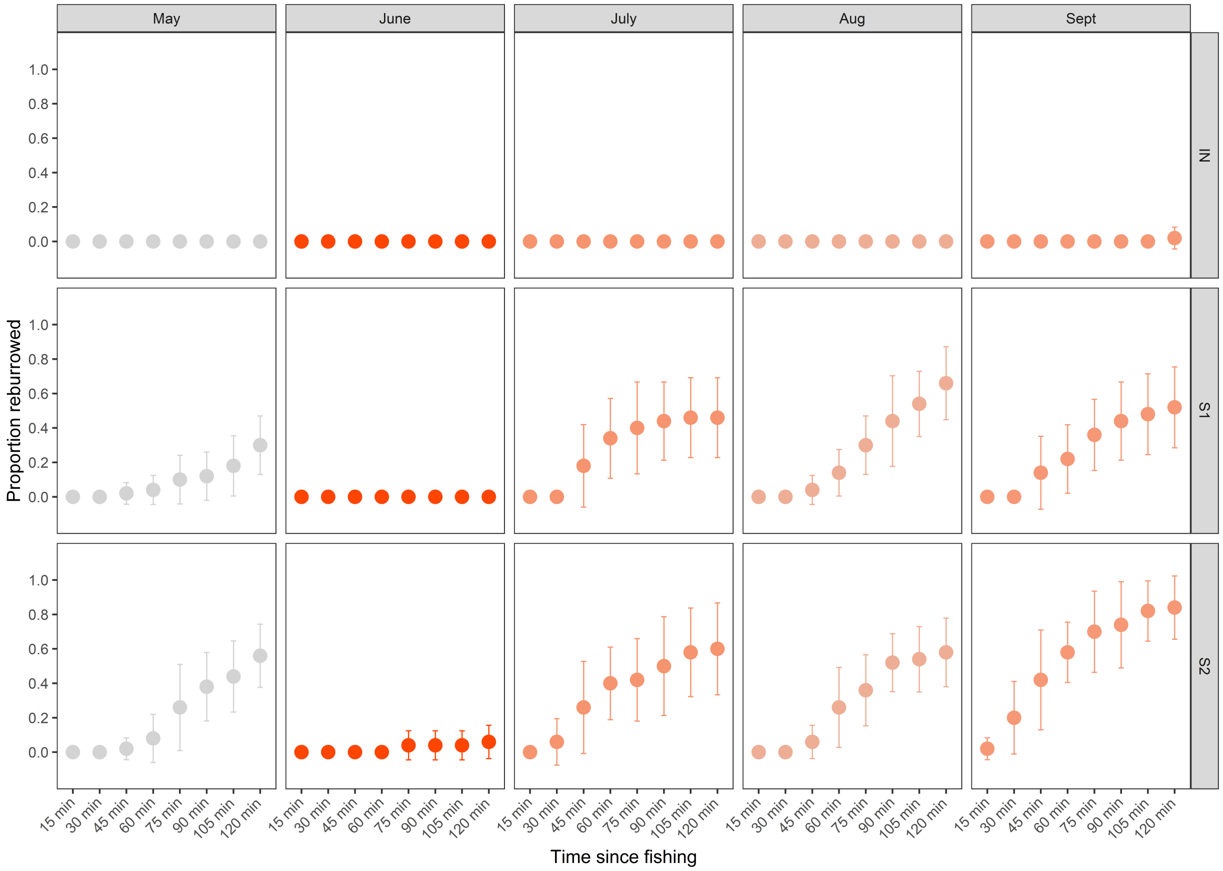

Figure SA1. Proportions of clams fully reburrowed during each 15 minute interval for the two hours after being returned to the sediment surface in each experiment and tide level. Data are means ± standard deviation. IN = intertidal; S1 = shallow subtidal; S2 = deeper subtidal.

Contrary to the intertidal, most of the clams in the two subtidal plots were able to reburrow within 2 hours of being tossed back, with the exception of clams in the June heatwave experiment (**Fig SA1**). Generally, clams in the two subtidal plots began reburrowing at 30-45 mins after being placed on the sediment surface, and 50-80% of the clams were reburrowed after two hours (**Fig. SA1**). Burrowing proportions after two hours generally appeared higher in the deeper subtidal plots compared to the shallow subtidal (although error bars generally show a fair degree of overlap, suggesting that any increased facilitation of reburrowing in deeper water is likely minimal). Once again, this is in line with the results of Ledoux et al.[4], who reported that clams placed back in water have a better chance of reburrowing and surviving after being fished, and that those placed in deeper water tend to reburrow slightly quicker, likely aided by increased downward water pressure.

Table SA1. Results of Bayesian generalized linear mixed models for the effects of experiment (May, June, July, August, September), time since fishing (15, 30, 45, 60, 75, 90, 105, 120 mins), and tide level (intertidal, shallow subtidal, deeper subtidal) on the proportion of reburrowed clams during the two hours following being placed back on the sediment surface on Day 1 of the experiments. Statistical model included Plot ID as a random variable to account for spatial effects and repeated measures over the two time points. Results were generated using the *Anova()* function from the 'car' package in R, which provides Wald chi-square test results for fixed effects. Bolded text denotes significant effects at $p \leq 0.05$.

	$\chi 2$	df	p-value
**(Intercept)**	**155.49**	**1**	**<0.0001**
**Experiment**	**17.94**	**4**	**0.0013**
**Time**	**14.21**	**7**	**0.0476**
**Tide level**	**10.89**	**2**	**0.0043**
Experiment × Time	29.22	28	0.4013
**Experiments × Tide level**	**48.58**	**8**	**<0.0001**
**Time × Tide level**	**208.23**	**14**	**<0.0001**
**Experiment × Time × Tide level**	**109.24**	**56**	**<0.0001**

While being placed in water aided reburrowing in clams for the May, July, August, and September experiments, this was not the case for the June heatwave experiment. Herein, the vast majority of clams failed to reburrow within two hours of being fished, with the only reburrowed clams being in the deeper subtidal (further support for placing clams in deeper water). This was mirrored by reburrowing proportions after 24- and 48 hours in the main analysis as well. This finding provides further support that the June heatwave exerted physiological stress on tossed back sub-legal clams, ultimately negating reburrowing and leading to eventual death.

References

1. Cheung, Y., Rabe-Hesketh, S., Dorie, V., Gelman, A., & Liu, J. A nondegenerate penalized likelihood estimator for variance parameters in multilevel models. *Psychometrika* **78**, 685–709. (2013).

2. Bolker, B. GLMM worked examples [online]. Accessed 1 October 2024 from https://bbolker.github.io/mixedmodels-misc/ecostats_chap.html#digression-complete-separation. (2018).

3. Fox, J., & Weisberg, S. *An R Companion to Applied Regression (3rd ed.)*. (Sage, Thousand Oaks, 2019).

4. Ledoux, T., Clements, J.C., Gallant, D., Sonier, R., & Miron, G. Burrowing behaviour of soft-shell clams (*Mya arenaria*) following human disturbance. *J. Exp. Mar. Biol. Ecol.* **565**, 151916. (2023).

5. Trueman, E.S. The burrowing process of *Dentalium* (Scaphopoda). *J. Zool.* 154, 19–27. (1968).

6. Clements, J.C., Ramesh, K., Nysveen, J., Dupont, S., & Jutfelt, F. Animal size and sea water temperature, but not pH, influence a repeatable startle response behaviour in a wide-ranging marine mollusc. *Anim. Behav.* **173**, 191–205. (2021).

7. Hubert, J., Booms, E., Witbaard, R., & Slabbekoorn, H. Responsiveness and habituation to repeated sound exposures and pulse trains in blue mussels. *J. Exp. Mar. Biol. Ecol.* **547**, 151668. (2022).
