# Supplementary material for: Fishing during extreme heatwaves alters ecological interactions and increases indirect fishing mortality in a ubiquitous nearshore system
Source: Commun Biol. 2025 May 12;8:735. doi: 10.1038/s42003-025-08158-w (PMC12069597; doi:10.1038/s42003-025-08158-w)
Supplement: Supplementary file 5 — Description of Additional Supplementary Files [file 42003_2025_8158_MOESM5_ESM.pdf]

Description of Additional Supplementary Files

File Name: Supplementary Data 1-10
Description: Source data for statistical analyses and figure generation (Supplementary Data 1-10). (.xlsx)

File Name: Supplementary Note
Description: Methodology, results, and interpretation of initial burrowing rates of sublegal sized clams every 15 minutes for two hours after being fished and tossed back on the sediment surface. (.docx)

File Name: Supplementary Code
Description: Annotated R code for statistical analysis and figure generation (including analysis in Supplementary Note and figures in Supplementary Information).
