# Supplementary material for: Fishing during extreme heatwaves alters ecological interactions and increases indirect fishing mortality in a ubiquitous nearshore system
Source: Commun Biol. 2025 May 12;8:735. doi: 10.1038/s42003-025-08158-w (PMC12069597; doi:10.1038/s42003-025-08158-w)
Supplement: Supplementary file 6 — Reporting Summary [file 42003_2025_8158_MOESM6_ESM.pdf]

nature portfolio

Corresponding author(s): Jeff C. Clements

Last updated by author(s): Mar 15, 2025

Reporting Summary

Nature Portfolio wishes to improve the reproducibility of the work that we publish. This form provides structure for consistency and transparency in reporting. For further information on Nature Portfolio policies, see our Editorial Policies and the Editorial Policy Checklist.

Statistics

For all statistical analyses, confirm that the following items are present in the figure legend, table legend, main text, or Methods section.

n/a	Confirmed	
☐	☒	The exact sample size (*n*) for each experimental group/condition, given as a discrete number and unit of measurement
☐	☒	A statement on whether measurements were taken from distinct samples or whether the same sample was measured repeatedly
☐	☒	The statistical test(s) used AND whether they are one- or two-sided<br>*Only common tests should be described solely by name; describe more complex techniques in the Methods section.*
☐	☒	A description of all covariates tested
☐	☒	A description of any assumptions or corrections, such as tests of normality and adjustment for multiple comparisons
☐	☒	A full description of the statistical parameters including central tendency (e.g. means) or other basic estimates (e.g. regression coefficient) AND variation (e.g. standard deviation) or associated estimates of uncertainty (e.g. confidence intervals)
☐	☒	For null hypothesis testing, the test statistic (e.g. *F*, *t*, *r*) with confidence intervals, effect sizes, degrees of freedom and *P* value noted<br>*Give P values as exact values whenever suitable.*
☐	☒	For Bayesian analysis, information on the choice of priors and Markov chain Monte Carlo settings
☐	☒	For hierarchical and complex designs, identification of the appropriate level for tests and full reporting of outcomes
☒	☐	Estimates of effect sizes (e.g. Cohen's *d*, Pearson's *r*), indicating how they were calculated

Our web collection on statistics for biologists contains articles on many of the points above.

Software and code

Policy information about availability of computer code

Data collection	n/a
Data analysis	All data were analyzed using existing available R code

For manuscripts utilizing custom algorithms or software that are central to the research but not yet described in published literature, software must be made available to editors and reviewers. We strongly encourage code deposition in a community repository (e.g. GitHub). See the Nature Portfolio guidelines for submitting code & software for further information.

Data

Policy information about availability of data

All manuscripts must include a data availability statement. This statement should provide the following information, where applicable:

- Accession codes, unique identifiers, or web links for publicly available datasets
- A description of any restrictions on data availability
- For clinical datasets or third party data, please ensure that the statement adheres to our policy

All supplementary data, code, and analyses are openly available through the Government of Canada's Open Data Portal here: https://open.canada.ca/data/dataset/1bf057da-8280-11ef-8cce-55cc7f028297. Files include supplementary figures (S1-S5) and tables (S1-S7), supplementary analysis (i.e., Day 1 reburrowing), annotated R code, original source data files used in analyses and figure generation (S1-S10 data), and a data dictionary with detailed explanations for each column header in each source data file. Source data files also serve as raw data files.

Research involving human participants, their data, or biological material

Policy information about studies with human participants or human data. See also policy information about sex, gender (identity/presentation), and sexual orientation and race, ethnicity and racism.

Reporting on sex and gender	n/a
Reporting on race, ethnicity, or other socially relevant groupings	n/a
Population characteristics	n/a
Recruitment	n/a
Ethics oversight	n/a

Note that full information on the approval of the study protocol must also be provided in the manuscript.

Field-specific reporting

Please select the one below that is the best fit for your research. If you are not sure, read the appropriate sections before making your selection.

☐ Life sciences ☐ Behavioural & social sciences ☒ Ecological, evolutionary & environmental sciences

For a reference copy of the document with all sections, see nature.com/documents/nr-reporting-summary-flat.pdf

Ecological, evolutionary & environmental sciences study design

All studies must disclose on these points even when the disclosure is negative.

Study description	This study documents the effects of human disturbance (clam digging) during an extreme heatwave on in situ predator-prey activity and indirect fishing mortality in a nearshore marine ecosystem. In a series of five field experiments, we mimicked clam digging during low tide and returned sub-legal sized soft-shell clams (Mya arenaria, 30-50mm shell length) back to the sediment surface in a fully-factorial experimental manner to test the independent and interactive effects of experimental trial (categorical fixed factor with five levels: May, June, July, August, September), predator treatment (categorical fixed factor with two levels: crab predator inclusion, crab predator exclusion), tide level (categorical fixed factor with three levels: intertidal, shallow subtidal, deeper subtidal), and time since fishing (categorical fixed factor with two levels: 24 h, 48 h) on the proportion of dead and reburrowed clams; plot ID (unique within each experiment) was included as a categorical random variable to account for random spatial effects and repeated measures across the two time points. We also conducted a secondary analysis to test more directly for heatwave effects by testing for effects of air temperature during fishing, experimental trial, tide level, time since fishing, and Julian date in a generalized additive mixed modeling approach. Proxies of predator activity (counts of crabs, and the presence/absence of omnivorous mudsnails) were also recorded in each experimental plot. Experiments deployed five replicate plots per predator treatment at each of the three tide levels during each of the five experiments; clam burrowing and mortality data, and proxies of predator activity, were collected 24 h and 48 h after fishing and returning clams to the sediment surface. While not included in the main paper, we also observed clam burrowing and mortality every 15 minutes for 2 hours (n = 8 time points) after returning the sub-legal clams to the sediment. We include these data analyzed separately from the main paper in a “Supplementary Analysis” file. Herein, the experimental design is the same as the above, except that the “time since fishing” factor has eight levels instead of two levels (15, 30, 45, 60, 75, 90, 105, and 120 mins).
Research sample	The experiments tracked groups of tagged, sub-legal sized (30-50 mm shell length) soft-shell clams, Mya arenaria. Sub-legal sized clams were chosen because the soft-shell clam fishery in our region is managed in part by a legal size limit, where clam diggers must toss back clams below 50mm shell length. Prior to being observed, clams were tagged by attaching fishing line tied to a metal washer to each individual clam using UV resin to make sure clams did not drift away from experimental plots and so that we knew which clams were our experimental clams within each plot. In addition to clams, we also counted the number of live crabs (Carcinus maenas and Cancer irroratus) found in each experimental plot during the 24 h and 48 h observation periods, and recorded whether or not the plots contained omnivorous mudsnails, Ilyanassa obsoleta.
Sampling strategy	Each experiment tracked groups of 5 clams per experimental plot (n = 150 clams per experiment; N = 750 clams total). Sample sizes were not computed in a predetermined manner, but were chosen based on logistical feasibility, minimizing impact to the clam population, reducing confounding effects of high clam densities within experimental plots, and based on previous work suggesting that these sample sizes are adequate. Sample sizes at the "plot" and "clam" level of replication were chosen based on previous work conducted at this site (Ledoux et al., 2023, J. Exp. Mar. Biol. Ecol., 565: 151916), with slightly increased sample sizes. Herein, we increased the number of "plot" replicates from n=4 in Ledoux et al. to n=5 plots (per predator treatment per tide level), and the number of "clam" replicates from n=3 to n = 5 clams per plot.
Data collection	After clams were placed back on the sediment surface, the number of clams that had burrowed or died was visually assessed and recorded every 15 minutes for 2 hours after fishing. The same metrics were also observed and recorded 24 and 48 hours after fishing, along with estimates of predator/scavenger activity. All data were initially recorded on paper and subsequently transferred to spreadsheets and saved as csv files. Data were then validated by a team member who did not enter the data, and further processed

	for analysis and figure generation.
Timing and spatial scale	Each experiment was conducted over the course of three days in each of the five months spanning May-September, 2024. Specific dates of the experiments were chosen based on predicted tidal amplitudes such that experiments coincided with low tides that were <0.3m relative to the lower low water large tide (LLWLT), as low tides higher than this range made accessing the intertidal zones difficult. Spatially, the experiment took place at a single experimental site in Kouchibouguac National Park, New Brunswick, Canada.
Data exclusions	No data were excluded from the study.
Reproducibility	Experiments were replicated over the course of five months. Consistent results were observed for the four non-heatwave experiments; however, the heatwave experiment was not replicated given that these were in situ field studies and other similar heatwaves did not occur during the study period.
Randomization	The spatial placement of plots within each tide level was fully randomized prior to each experiment using Microsoft Excel. Individual clams were placed haphazardly into each plot prior to experimental observations.
Blinding	Given the distinct visual differences between predator inclusion and exclusion cages and the obvious nature of each tide level, blinded observations were not possible for these experiments. However, observations of clam burrowing were made by at least two field team members to avoid individual researcher bias. If there were discrepancies between team members regarding whether a clam was burrowed, an additional team member would provide their assessment and the team would collectively determine if a clam was or was not considered burrowed.

Did the study involve field work? ☒ Yes ☐ No

Field work, collection and transport

Field conditions	All experiments were conducted on clear, sunny days with minimal overcast and wind. Air temperatures during each of the five experiments varied
Location	Loggiecroft Beach, Kouchibouguac National Park, New Brunswick, Canada (46.835087°N, 64.932245°W)
Access & import/export	Habitat access and clam collection were conducted in accordance with federal regulations under DFO Section 52 License number SG-RHQ-24-076 (request no. 8) and Parks Canada Agency Research and Collection Permit number KOUCHNP-2024-458201.
Disturbance	Digging clams causes disturbance to the sediment surface and can create holes in the sediment. Holes were always refilled once clams were collected. All legal-sized clams (>50mm shell length) and clams <30mm were immediately returned to the subtidal sediment surface to maximize survival (as per Ledoux et al., 2023, J. Exp. Mar. Biol. Ecol., 565: 151916).

Reporting for specific materials, systems and methods

We require information from authors about some types of materials, experimental systems and methods used in many studies. Here, indicate whether each material, system or method listed is relevant to your study. If you are not sure if a list item applies to your research, read the appropriate section before selecting a response.

Materials & experimental systems

n/a	Involved in the study	
☒	☐	Antibodies
☒	☐	Eukaryotic cell lines
☒	☐	Palaeontology and archaeology
☐	☒	Animals and other organisms
☒	☐	Clinical data
☒	☐	Dual use research of concern
☒	☐	Plants

Methods

n/a	Involved in the study	
☒	☐	ChIP-seq
☒	☐	Flow cytometry
☒	☐	MRI-based neuroimaging

Animals and other research organisms

Policy information about studies involving animals; ARRIVE guidelines recommended for reporting animal research, and Sex and Gender in Research

Laboratory animals	n/a
Wild animals	The experiments were conducted using sub-legal sized soft-shell clams, Mya arenaria (30-50mm shell length). The animals were fished by locating surface sediments with high concentrations of visible siphon holes and digging the clams with a shovel. Live clams obtained during digging were placed in a 5 gallon bucket of fresh seawater (changed every 45-60 mins) until tagging; the clams were

	then exposed to air for 30-60 mins prior to experimentation. After each of the five experiments, the fishing line and washers were removed from each of the clams (whether dead or alive) and the clams were immediately released back to their natural habitat.
Reporting on sex	n/a
Field-collected samples	The study involved field-collected samples, with experiments being conducted in situ to mimick common clam digging practices in this region. Upon being fished, clams were kept in a 5 gal bucket full of seawater until 150 sub-legal clams were obtained; seawater int he bucket was changed every 45-60 mins. Once 150 sub-legal clams were caught, the clam shell was dried with a paper towel and the fishing line with metal washers were affixed to each clam with UV resin; clams were exposed to air for ~30-60 mins while UV resin dried. The clams were then placed in their respective experimental plots after which data collection began.
Ethics oversight	Sampling did not require ethics approval from the Canadian Council of Animal Care (CCAC), as the study species is not considered under their purview.

Note that full information on the approval of the study protocol must also be provided in the manuscript.

Plants

Seed stocks	n/a
Novel plant genotypes	n/a
Authentication	n/a
